# Follow-up of women after gynecological cancer treatment

**DOI:** 10.61622/rbgo/2025FPS3

**Published:** 2025-03-28

**Authors:** Agnaldo Lopes da Silva, Mariana Seabra Leite Praça, Matheus Eduardo Soares Pinhati, Laura Guimarães Castro, Renato Moretti-Marques, Angélica Nogueira-Rodrigues, Eduardo Batista Cândido

**Affiliations:** 1 Faculdade de Medicina Universidade Federal de Minas Gerais Belo Horizonte MG Brazil Faculdade de Medicina, Universidade Federal de Minas Gerais, Belo Horizonte, MG, Brazil.; 2 Faculdade de Medicina Universidade Federal de Minas Gerais Belo Horizonte MG Brazil Faculdade de Medicina, Universidade Federal de Minas Gerais, Belo Horizonte, MG, Brazil.; 3 Faculdade de Medicina Universidade Federal de Minas Gerais Belo Horizonte MG Brazil Faculdade de Medicina, Universidade Federal de Minas Gerais, Belo Horizonte, MG, Brazil.; 4 Faculdade de Medicina Universidade Federal de Minas Gerais Belo Horizonte MG Brazil Faculdade de Medicina, Universidade Federal de Minas Gerais, Belo Horizonte, MG, Brazil.; 5 Hospital Israelita Albert Einstein São Paulo SP Brazil Hospital Israelita Albert Einstein, São Paulo, SP, Brazil.; 6 Faculdade de Medicina Universidade Federal de Minas Gerais Belo Horizonte MG Brazil Faculdade de Medicina, Universidade Federal de Minas Gerais, Belo Horizonte, MG, Brazil.; 7 Faculdade de Medicina Universidade Federal de Minas Gerais Belo Horizonte MG Brazil Faculdade de Medicina, Universidade Federal de Minas Gerais, Belo Horizonte, MG, Brazil.

## Abstract

•The population of female cancer survivors has increased over the last few years, highlighting the importance of appropriate follow-up of these patients.

•The main objective of long-term follow-up for patients treated for cancer is the early detection of recurrences, whether local, lymph node or distant metastases.

•Symptom assessment and physical examination play an important role in the follow-up of patients treated for gynecological neoplasms.

•The use of laboratory or imaging tests to detect recurrence in asymptomatic patients should be based on evidence that it improves survival or provides less morbid treatments, also considering cost and availability.

**Recommendations**

•The frequency of follow-up of patients varies according to the neoplasm, stage, treatment and risk of recurrence.
•Patients should be advised about signs and symptoms suggestive of recurrence, which vary according to the neoplasm treated.
•Imaging tests are indicated in cases of clinical suspicion of recurrence of gynecological neoplasms.
•The use of CA-125 is not routinely recommended in the follow-up of endometrial cancer. However, in ovarian neoplasms in which CA-125 was elevated before primary treatment, its use may have prognostic value.
•Germ cell tumors of the ovary have specific markers that can be used during follow-up.
•Although evidence is limited, cytology can be performed annually in the follow-up of patients treated for neoplasms of the cervix, vulva, and vagina. Prior radiotherapy increases the false-positive rate.
•Patients treated with radical surgeries of the vulva and vagina should be monitored by professionals experienced in the treatment and physical examination of these neoplasms.

## Background

Each year, gynecological cancers are responsible for approximately 1.5 million new diagnoses and more than 680,000 deaths worldwide.^(1)^ In Brazil, more than 32,000 new cases are expected in 2024.^(2)^ Numerous advances in gynecological oncology over the last few years have increased the survival of these patients, who now consist of a larger and longer-lived population of survivors.^(3)^

Based on the idea that early detection of recurrences can lead to less invasive treatments with a higher survival rate, providing adequate follow-up for these women is essential. Furthermore, it is crucial that the fear of relapse and anxiety preceding follow-up appointments after treatment do not impact quality of life and use of health services.^(4)^ The costs and availability of resources to perform these tests should also be considered.^(5,6)^ Nevertheless, many studies report that cancer survivors often do not receive appropriate care.^(7,8)^

Despite advances in treatments and increased survival rates for surviving patients, the available information regarding follow-up after treatment for gynecological neoplasms lacks scientific evidence, and is predominantly derived from retrospective studies and expert opinions.^(3)^ Some follow-up models currently adopted may be inflexible and do not provide personalized, cost-effective care to patients.^(9)^ Therefore, informing gynecologists about the frequency, assessment, and examinations required for follow-up of lower genital tract cancers is essential to optimize the care provided to these patients.

### Endometrial cancer

Endometrial cancer was responsible for 420,242 new cases worldwide in 2022.^(1)^ In Brazil, approximately 7,840 new cases were estimated for 2024.^(2)^ Most patients are diagnosed in the early stages of the disease, from an early symptomatic picture characterized by metrorrhagia or postmenopausal vaginal bleeding (83%). Despite the high survival rate, endometrial cancer can present considerable recurrence rates, which affect 2% to 15% of patients with the neoplasia in early stages and reach 50% in patients with the disease in more advanced stages or with more aggressive histological conditions.^(5)^

For most patients, recurrence is symptomatic and occurs in the first three years after primary treatment. Even in patients with distant recurrence, symptoms such as cough, pain, lethargy, weight loss and headache are present in 70% of cases.^(5)^ After primary treatment, the National Comprehensive Cancer Network (NCCN)^(10)^ highlights the importance of verbal and written recommendations about symptoms that indicate potential recurrence of the disease, so that patients seek medical evaluation. The main symptoms include: vaginal bleeding, decreased appetite, weight loss, pain (in the pelvis, abdomen, hip or lower back), cough, dyspnea and edema (abdominal and lower limbs).^(10)^

There is no high-quality evidence associating follow-up strategies after primary treatment with favorable outcomes and there is no consensus on which tests should be offered for follow-up of survivors after endometrial cancer.^(11)^ A multicenter review examined the efficiency of follow-up methods in 254 patients with high-grade disease, revealing that symptoms led to the detection of most recurrences (56%), followed by physical examination (18%), computed tomography (CT) (15%), CA-125 measurement (10%) and performance of oncotic cytology (1%).^(12)^

Physical examination is considered the most consistent method of monitoring endometrial cancer and should include speculum examination and vaginal examination for pelvic and retrovaginal evaluation. The NCCN recommends the follow-up of patients with a physical examination and detailed history every three to six months for the first two to three years, as the risk of recurrence is higher during this period. Thereafter, up to the fifth year, follow-up should be performed every six to 12 months, and after the fifth year, the evaluation should be annual.^(10)^ The European Society for Medical Oncology (ESMO) guidelines adjust endometrial cancer surveillance by risk factors. Patients with locally advanced disease, stages III-IV, high-grade tumors, lymph node involvement, and non-endometrioid histological types are considered high-risk. In this group, the recommendation is to perform physical and gynecological examinations every three months during the first three years and every six months thereafter, up to the fifth year after primary treatment. In the low-risk group, follow-up is suggested every six months during the first two years and annually until the fifth year (Chart 1). In this group, remote follow-up by telephone contact may be an alternative if the patient has received adequate education on the warning signs and symptoms of recurrence.^(13)^

**Chart 1:** Recommendations for endometrial cancer

Guideline	NCCN	ESMO
Examination and review of symptoms	Every 3-6 months for 2 or 3 years, then every 6 months until the 5^th^ year. Annually thereafter.	Low risk: every 6 months (consider telephone contact) for 2 years. Annually thereafter. High risk: every 3 months for 3 years, then every 6 months until the 5^th^ year. Annually thereafter.
CT scan of abdomen and pelvis	Only in cases of advanced disease at diagnosis, every 6 months for the first 3 years, every 6-12 months for an additional 2 years.	Only in the high-risk group every 6 months for the first 3 years, then individually thereafter.
Other imaging tests*	Only if there is clinical suspicion of recurrence.	Only if there is clinical suspicion of recurrence.
CA-125	Not recommended for routine use.	Not recommended for routine use.
Oncotic cytology	Not recommended for routine use.	Not recommended for routine use.

*Imaging tests (chest X-ray, chest CT, abdominal/pelvic MRI, and PET-CT) should be considered in selected patients, as clinically indicated. NCCN: National Comprehensive Cancer Network; ESMO: European Society for Medical Oncology; CT: computed tomography; MRI: magnetic resonance imaging; PET-CT: positron emission tomography-computed tomography Source: Adapted from Salani et al. (2017)^(^^5^^)^ and National Comprehensive Cancer Network.^(^^10^^)^

Imaging tests should not be routine in the follow-up of patients with endometrial cancer. Their indication should be guided by the patient’s symptoms, risk assessment, and clinical suspicion of recurrence of the neoplasm or metastatic disease. Therefore, imaging studies such as chest X-ray and CT, and abdominal and pelvic magnetic resonance imaging (MRI) should only be performed if the patient is symptomatic or there is a clinical indication.^(10)^ The ESMO endorses this recommendation.^(13)^ Routine CT scans may be considered (e.g., every six months for the first three years, and the request should be assessed individually after that) in high-risk groups, especially those with lymph node involvement.

As previously mentioned, there may be an association between elevated CA-125 levels and recurrence of endometrial cancer. This marker increases in more than 50% of patients in advanced stages or more aggressive histological grades of the disease. As the association between recurrence and elevated levels of CA-125 has not been demonstrated in early stages or less aggressive histological grades^(14)^, this marker should not be routinely used as a marker of recurrence.

Although most recurrences of the disease occur in the vaginal vault, the performance of oncotic cytology for follow-up is controversial. Abnormal cytology has a sensitivity of 40% and a specificity of 88% for the detection of vaginal recurrence of the disease.^(15)^ Therefore, according to the NCCN and ESMO, oncotic cytology is not useful for the diagnosis of local recurrences and should not be routinely performed in asymptomatic patients.^(10,13)^

### Ovarian cancer

In 2022, ovarian cancer was responsible for 324,398 new diagnoses and 206,839 deaths worldwide, which represents most deaths from gynecological tumors.^(1)^ The lack of effective screening methods and the presence of nonspecific symptoms lead to late diagnosis in approximately 75% of patients, directly impacting the average five-year survival rate. More than 70% of patients with ovarian cancer will experience tumor recurrence, which justifies the search for optimal and more effective surveillance after treatment.^(5)^

### High-grade serous carcinomas

High-grade serous carcinoma accounts for approximately 70%-80% of all ovarian malignancies.^(16)^ Most high-grade serous carcinomas are diagnosed at an advanced stage (stage III or IV), when survival rates are 41% and 20% for stages III and IV, respectively.^(17)^ Although approximately 80% of patients respond completely to primary treatment, the recurrence rate is high. The recurrence rate is 25% of cases in the early stages of the disease, and reaches more than 80% of cases in advanced stages.^(5)^

Between 26% and 50% of ovarian tumor recurrences occur in the pelvis;^(5)^ approximately half of cases are detected through symptoms and 60% through physical examination, which shows the relevance of this evaluation for detecting tumor progression.^(18)^ However, physical examination has variable reproducibility (15% to 78%) and may not identify recurrences in extrapelvic regions such as retroperitoneal lymph nodes, abdominal organs, liver or lungs.^(5)^

At diagnosis, the tumor marker CA-125 is elevated in approximately 80% of epithelial tumors, and it has a sensitivity of 62%-94% and specificity of 91%-100% for detecting recurrence.^(5)^ Although the EORTC 55955 randomized trial presents limitations, an improvement in survival has not been found when treating recurrences based solely on CA-125 levels compared to clinical suspicion.^(19)^ However, both the NCCN and the American National Cancer Institute (NCI) recommend requesting CA-125 as part of the follow-up consultation for ovarian tumors, even in asymptomatic patients. Preclinical serum CA-125 elevation may precede clinical recurrence by two to five months.^(20)^ Currently, with the availability of more sensitive imaging methods, the possibility of performing complete secondary cytoreduction, and the emergence of targeted therapies that improve the results of ovarian cancer treatment, the benefit of following CA-125 dosage remains to be defined.^(21)^ Although the use of human epididymal protein 4 (HE4) as an independent predictive biomarker for monitoring and tracking recurrence is promising, more robust evidence is needed to include the request for this marker in clinical practice.^(22)^

The sensitivity of imaging tests to detect recurrence of serous ovarian tumors is limited.^(23)^ Magnetic resonance imaging has a sensitivity of 62%-91% and a specificity of 40%-100%, being superior to CT for assessing operability in cases of recurrence.^(24)^ Although the PET-CT has high sensitivity and specificity (89% and 90%, respectively), as evidence demonstrating improved survival is still lacking, it is recommended only in the presence of symptoms and changes in the physical examination or in CA-125 levels that suggest recurrence (Chart 2).^(20)^

**Chart 2:** Recommendations for high- and low-grade serous ovarian carcinomas

Time since completion of primary therapy	Up to 2^nd^ year	3^rd^ year	4^th^ and 5^th^ year	After 5^th^ year
Examination and review of symptoms	Every 3-4 months	Every 4-6 months	Every 6 months	Annually
CA-125^a^	Every 3-4 months	Every 4-6 months	Every 6 months	Annually
Imaging tests^b^	Not recommended for routine use			
Suspected recurrence or metastasis	Abdominal/pelvic MRI or PET-CT ± CA-125			

*Recommended if elevated before treatment. **Imaging tests (chest X-ray, chest CT, abdominal/pelvic MRI, and PET-CT) should be considered in selected patients, as clinically indicated. Recommended if elevated before treatment; CT: computed tomography; MRI: magnetic resonance imaging; PET-CT: positron emission tomography-computed tomography Source: Adapted from Salani et al. (2017)^(^^5^^)^ and the National Comprehensive Cancer Network.^(^^20^^)^

The follow-up of patients with BRCA1 and BRCA2 mutations should be longer due to the risk of breast cancer. These survivors should be referred to referral services for monitoring patients at high risk for breast tumors.^(21)^

### Low-grade serous carcinomas

Low-grade serous carcinoma represents less than 5% of epithelial ovarian cancers. Although these tumors are associated with a more indolent disease, they usually also present in advanced stages at diagnosis. Approximately 60% of low-grade serous carcinomas are also associated with borderline tumors.^(20)^ Follow-up recommendations are the same as those for high-grade serous carcinomas (Chart 2).

### Borderline tumors

Approximately 65%-70% of borderline tumors are serous, which corresponds to approximately 15%-20%^(25)^ of all ovarian serous neoplasms.^(26-28)^ In the vast majority of patients, serous borderline tumors are confined to the ovary at the time of diagnosis and are bilateral in up to 50% of cases.^(29)^ Approximately 70% of cases are diagnosed at stage I.^(30)^ Mucinous tumors are another common histological type, accounting for 11% of borderline tumors.^(31)^ The recurrence rate of borderline tumors ranges from 5% to 8%, and progression to invasive tumors occurs in approximately 2% of cases. In general, 70% of recurrences will occur after five years and 30% after ten years.^(25,32)^ The risk of recurrence is higher in patients who underwent conservative surgeries that preserved one or both ovaries, and is approximately six times higher in women who underwent ovarian cystectomy.^(32)^

Current follow-up guidelines for these tumors go beyond the recommendations for invasive ovarian cancer. Depending on the extent of surgery and the stage of the disease, a physical examination is suggested every three to six months, including pelvic examination, CA-125 measurement (if initially elevated) and pelvic ultrasound for patients who underwent fertility-preserving surgery.^(5,20)^

For patients diagnosed at stage I who underwent bilateral salpingo-oophorectomy, annual follow-up is indicated, since there is no benefit from closer surveillance. For patients who have undergone fertility-preserving surgery, such as unilateral salpingo-oophorectomy or cystectomy, the risk of recurrence ranges from 5% to 7%.^(25,32,33)^ Current follow-up recommendations for these patients include serial pelvic ultrasound, with or without assessment of tumor markers. Although hysterectomy with bilateral salpingo-oophorectomy is recommended for patients with defined offspring, there are no studies indicating that this intensive follow-up or even that hysterectomy improves the prognosis for women with borderline tumors.^(5,20)^

In patients with advanced borderline tumors (stage II-IV), annual review of symptoms and physical examination with or without tumor markers (if initially elevated) seems appropriate (Chart 3).^(20)^

**Chart 3:** Recommendations for borderline ovarian cancers

Time since completion of primary therapy	Up to 5^th^ year	After 5^th^ year
Examination and review of symptoms	3-12 months	Annually
Ultrasound	Patients who have preserved fertility	Only if clinically suspected
Imaging tests*	Only if clinically suspected	Only if clinically suspected
CA-125 or other markers	Recommended if elevated before treatment	Recommended if elevated before treatment
Cytology	Not recommended	Not recommended

*Imaging exams (chest radiograph, chest CT, abdominal/pelvis MRI, and PET-CT) should be considered in selected patients, as clinically indicated; CT: computed tomography; MRI: magnetic resonance imaging; PET-CT: positron emission tomography-computed tomography Source: Adapted from the National Comprehensive Cancer Network.^(^^20^^)^

When clinical or laboratory recurrence is suspected, CT of the abdomen and pelvis is recommended to assess the extent of the disease. As most women with borderline tumors can be treated with additional surgery, attention to symptoms or abnormalities on physical examination is important. There is no evidence that routine radiographic surveillance with CT is beneficial.^(20)^

### Germ cell tumors

Malignant germ cell tumors of the ovary represent 2.6% of all ovarian tumors. They include the following subtypes: dysgerminomas, immature teratomas, embryonal tumors, and endodermal sinus tumors. They are predominant in women with an average age of 16-20 years.^(34,35)^ They are usually unilateral and diagnosed at stage I. Therefore, fertility-preserving surgeries with or without adjuvant therapy can be performed for treatment. Recurrences are rare (15%-25%) and usually occur in the first two years after primary treatment.^(20)^

These tumors can produce serological tumor markers that aid in diagnosis and post-treatment follow-up. Alpha-fetoprotein (α-FP) can be produced by endodermal sinus tumors, embryonal tumors, polyembryomas, and immature teratomas. Human chorionic gonadotropin (hCG) can be produced by choriocarcinomas, embryonal tumors, polyembryomas, and, in low levels, some dysgerminomas. Lactate dehydrogenase (LDH) may be a marker for dysgerminomas.^(20)^

According to the NCCN recommendations, the follow-up of patients with germ cell tumors should include physical examination and analysis of tumor marker levels, in addition to imaging tests. In dysgerminomas, follow-up with physical examination and analysis of biomarkers should be performed every two to three months in the first year, three to four months in the second year, six months up to the fifth year, and annually thereafter. Imaging tests (CT scans of the abdomen and pelvis) should be requested every three to four months in the first year, six months in the second year, and only if clinically indicated thereafter. In other subtypes of germ cell tumors, physical examination and analysis of tumor biomarkers should be performed every two months in the first two years, four to six months in the third year, six months in the fourth and fifth years, and annually thereafter. Imaging tests (CT scans of the chest, abdomen, and pelvis) should be requested every three to four months in the first year, four to six months in the second year, six to twelve months from the third to the fifth year, and thereafter only if clinically indicated (Chart 4). This active surveillance proposal is similar to that suggested by the ESMO, especially in patients undergoing fertility-preserving surgery and without adjuvant treatment. The follow-up schedule is meticulous and patient compliance is extremely important. Patients must be counseled on pregnancy prevention during the first two years after initial diagnosis, the period in which most recurrences occur.^(20,36)^

**Chart 4:** Recommendations for germline ovarian tumors

Time since completion of primary therapy	1^st^ year	2^nd^ year	3^rd^ year	4^th^ and 5^th^ years	After 5^th^ year
**Dysgerminomas**					
Physical examination and analysis of tumor markers	Every 2-3 months	Every 3-4 months	Every 6 months	Every 6 months	Annually
Imaging tests*	Every 3-4 months	Every 6 months	Annually	Annually	Only if clinically suspected
**Non-dysgerminomas**					
Physical examination and analysis of tumor markers	Every 2 months	Every 2 months	Every 4-6 months	Every 6 months	Annually
Imaging tests^**^	Every 3-4 months	Every 4-6 months	Every 6-12 months	Every 6-12 months	Only if clinically suspected

*CT scan of the abdomen/pelvis; **CT scan of the chest/abdomen/pelvis; CT: computed tomography Source: Adapted from the National Comprehensive Cancer Network.^(^^20^^)^

### Stromal tumors

Malignant sex cord tumors are rare and account for 7% of ovarian malignancies. Typically associated with a good prognosis, they include granulosa cell tumors (the most common) and Sertoli-Leydig cell tumors. They can also produce tumor markers, such as estradiol, inhibin, anti-Müllerian hormone, and testosterone. Granulosa cell tumors may have an indolent course with the possibility of late recurrence, especially within a period of four to six years after primary treatment. Recurrences tend to occur in the upper abdomen (55%-70%) and pelvis (30%-45%), and response rates are generally favorable, reaching 63%-80%.^(36,37)^

The NCCN and the European Society of Gynecological Oncology (ESGO)-ESMO recommend the follow-up of patients with malignant sex cord tumors with physical examination and tumor marker analysis, if applicable. Imaging tests should be reserved for patients with symptoms, elevated biomarkers, or suspicious findings on physical examination. The frequency of follow-up with physical examination and request for tumor biomarker analysis should be based on the tumor stage; every six to 12 months for early stages and every four to six months for high-risk diseases.^(25)^ Prolonged follow-up is recommended for granulosa cell tumors because of the high possibility of late recurrence (Chart 5).^(38-40)^

**Chart 5:** Recommendations for ovarian stromal tumors

Time since completion of primary therapy	Up to 2^nd^ year	After 2 years
Physical examination	Only if clinically suspected, with frequency based on disease staging: Early stage/low-risk disease: every 6-12 months High-risk disease: every 4-6 months	Only if clinically suspected, with frequency based on disease staging: Early stage/low-risk disease: every 6-12 months High-risk disease: every 4-6 months
Analysis of tumor markers inhibin B and AMH	Only if clinically suspected and if applicable, with frequency based on disease staging: Early stage/low-risk disease: every 6-12 months High-risk disease: every 4-6 months	Only if clinically suspected and if applicable, with frequency based on disease staging: Early stage/low-risk disease: every 6-12 months High-risk disease: every 4-6 months
Imaging tests*	Reserved for patients with symptoms, elevated biomarkers, or suspicious findings on physical examination	Reserved for patients with symptoms, elevated biomarkers, or suspicious findings on physical examination

*Chest X-ray, CT of the chest/abdomen/pelvis, MRI, PET-CT; AMH: anti-Müllerian hormone; CT: computed tomography; MRI: magnetic resonance imaging; PET-CT: positron emission tomography-computed tomography Source: Adapted from the National Comprehensive Cancer Network.^(^^20^^)^

### Cervical cancer

In 2022, there were 661,021 new cases of cervical cancer and 348,189 deaths related to this disease worldwide.^(1)^ In Brazil, approximately 17,010 new cases are estimated annually for 2024.^(2)^ Cervical cancer predominantly affects younger individuals compared to other gynecologic cancers, with a median age of 50 years at diagnosis. Approximately 50% of patients are diagnosed at stage I, with a five-year survival rate of over 90%. However, recurrence rates for these patients are high, ranging from 10% to 20%, with the majority occurring within the first two to three years after initial treatment.^(41,42)^ Many of these patients with early-stage disease are cured and have a long life expectancy after treatment, with additional concerns about fertility preservation. Although chemoradiotherapy is usually the ideal treatment for locally advanced disease and can be curative, it results in significant sequelae and does not preserve fertility or ovarian function.^(43)^ The average survival ranges from eight to 53 months in asymptomatic patients and from eight to 38 months in symptomatic patients.^(41)^

History assessment and physical examinations are recommended every three to six months for the first two years, every six to 12 months for an additional three to five years, and annually thereafter. High-risk patients (advanced stage, treated with primary chemoradiotherapy or surgery plus adjuvant therapy) may require more frequent evaluations than low-risk patients (early stage, treated with surgery alone, without adjuvant therapy).^(44,45)^ However, this follow-up routine identifies less than 36% of recurrence cases during this period.^(42)^ Therefore, it is essential to inform patients about signs and symptoms that suggest recurrence, such as pelvic pain, lymphedema, vaginal bleeding, and genitourinary symptoms, present in 46% to 95% of cases and responsible for seeking care outside the usual recommended routine (Chart 6).^(5)^

**Chart 6:** Recommendations for cervical neoplasms

Time since completion of primary therapy	Up to 2^nd^ year	From 3º ao 5º year	After 5^th^ year
Examination and review of symptoms			
Low risk	Every 6-12 months	Annually^*^	Annually^*^
High risk^**^	Every 3 months	Every 6 months	Annually^*^
Cytological examination	Annually^***^		
Imaging tests^****^			
Stage I			
Without fertility preservation	Only if there is clinical suspicion of recurrence.^*****^ For patients with FIGO stage IB3 or who required postoperative adjuvant radiotherapy or chemoradiotherapy due to high-risk factors,^******^ PET-CT of the neck/chest/abdomen/pelvis/inguinal region may be performed 3-6 months after completion of treatment.^********^		
With fertility preservation	Contrast-enhanced MRI of the pelvis 6 months after surgery and then annually for 2-3 years. Other imaging tests only if there is clinical suspicion of recurrence.^*****^		
Stage II-IV	PET-CT of the neck/chest/abdomen/pelvis/inguinal region (preferred) or contrast-enhanced CT of the chest/abdomen/pelvis within 3-6 months after completion of treatment. ^********^ Contrast-enhanced MRI of the pelvis within 3-6 months after completion of therapy. Other imaging tests only if there is clinical suspicion of recurrence.^*******^		
Stage IVB or recurrence	CT, MRI, or PET-CT to assess response or determine additional treatment.		
Suspected recurrence or metastasis	PET-CT of the neck/chest/abdomen/pelvis/inguinal. Consider MRI of the pelvis.		

^*^May be followed by a gynecologic oncologist or general gynecologist. ^**^ High risk is defined as advanced stage or high-risk histologies. ^***^Insufficient evidence for detection of cancer recurrence, but may have value in detection of lower genital tract cancer and in immunocompromised patients. ^****^MRI is performed with and without contrast, and CT is performed with contrast unless contraindicated. Contrast is not required for screening chest CT. ^*****^These factors include abnormal findings on physical examination or pelvic, abdominal, or pulmonary symptoms. ^******^Risk factors may include positive nodes, positive parametria, positive margins, or local cervical factors (according to the Sedlis Criteria). ^*******^If the first post-treatment FDG-PET-CT is indeterminate, consider repeating it in three months. ^********^These factors may include abnormal findings on physical examination, such as a palpable mass or adenopathy, or new pelvic, abdominal, or pulmonary symptoms; FIGO: International Federation of Gynecology and Obstetrics; CT: computed tomography; MRI: magnetic resonance imaging; PET-CT: positron emission tomography-computed tomography; FDG-PET-CT: fluorodeoxyglucose PET-CT Source: Adapted from the National Comprehensive Cancer Network.^(^^45^^)^

It is crucial to perform detailed physical examinations during follow-up visits, including speculum examination and bimanual and rectovaginal examination, in addition to evaluating areas susceptible to lesions caused by human papillomavirus (HPV), such as the vagina, vulva, and anus. This practice detects between 29% and 75% of recurrent lesions or metastases in asymptomatic patients.^(5)^

While the latest edition of the ESGO-ESMO guideline (updated in 2023) does not recommend performing cytopathology, the NCCN recommends performing it annually.^(44,45)^ In patients treated with primary radiotherapy, the incidence of abnormal oncotic cytology reaches 34%, and most findings are atypical squamous cells of undetermined significance (ASCUS).^(5)^

The use of imaging tests should be based on the presence of symptoms or abnormal findings on physical examination. In a systematic review of 17 retrospective studies, asymptomatic recurrent disease was detected by physical examination in 29%-71%, chest radiography in 20%-47%, CT in 0%-34%, and vaginal vault cytology in 0%-17% of patients.^(46)^ However, if recurrent disease is suspected, imaging tests are indicated for a more accurate assessment of the extent of the disease. In this case, PET-CT stands out by its high accuracy, with sensitivity of around 86% and specificity of 87%.^(5)^

### Vulvar cancer

Responsible for 47,342 new diagnoses in 2022 and 18,579 deaths worldwide in 2022, vulvar cancer is relatively rare, representing less than 1% of all tumors in women.^(1)^ Radical vulvectomy and inguinofemoral lymphadenectomy have been the standard treatment. However, more recent treatments include adjuvant chemoradiotherapy for large primary tumors involving the urethra, vagina, or anus and the incorporation of sentinel lymph node assessment. Inguinal and/or femoral lymph node involvement is the most significant prognostic factor for survival in patients with vulvar cancer.^(5)^ Reported five-year survival ranges from 70% to 93% for patients with negative lymph nodes and from 25% to 41% for those with positive lymph nodes.^(47)^

Most vulvar cancer recurrences occur within the first year after definitive treatment. However, in a case series, nearly 10% of patients had a second malignancy diagnosed five years after initial treatment. In the GROINSS-V study, the local recurrence rate was 27.5% at five years and 39.5% after ten years of primary treatment.^(48)^

After treatment with curative intent, the ESGO-ESMO suggests more rigorous follow-up: initial follow-up six to eight weeks after completion of treatment and every three to four months during the first two years. From the third to the fifth year, follow-up may be semiannual or annual. Long-term surveillance may be appropriate in individuals with persistent predisposing vulvar disease or due to treatment-related secondary events.^(49)^ The Society of Gynecologic Oncology (SGO) recommends monitoring patients in early stages (I and II) every six months during the first two years and annually thereafter. For advanced stage disease (III and IVA), follow-up is done every three months for the first two years, then every six months between third and fifth years, and annually thereafter (Chart 7).^(5)^

**Chart 7:** Recommendations for neoplasms of the vulva and vagina

Time after primary treatment	1^st^ year	2^nd^ year	From 3^rd^ to 5^th^ year	After 5^th^ year
Low risk	Every 6 months	Every 6-12 months	Annually^*^	Annually^*^
High risk^**^	Every 3 months	Every 3 months	Every 6 months	Annually^*^
Pap smear/cytology test	Annually^***^			
Imaging tests^****^	Insufficient evidence for routine use			
Suspected recurrence	CT or PET-CT			

^*^May be followed by a gynecologic oncologist or general gynecologist. **High risk is defined as advanced stage or high-risk histologies. ***Insufficient evidence for detecting cancer recurrence, but may be valuable in detecting lower genital tract neoplasia and in immunocompromised patients. ****May include chest radiography, computed tomography, PET-CT, magnetic resonance imaging, and ultrasound. CT: computed tomography; PET-CT: positron emission tomography-computed tomography Source: Adapted from Oonk et al. (2023).^(^^49^^)^ and Nout et al. (2023).^(^^51^^)^

Evaluations should include a review of symptoms and a complete physical examination of the vulva, adjacent skin, and inguinal lymph node. Although evidence is limited, cytologic examination may be done annually.^(5)^

Routine use of imaging tests in the absence of symptoms or examination findings should also be avoided, since most recurrences are easily detected by physical examination. If recurrence is suspected, CT or PET-CT may be requested, as well as colposcopy and targeted vulvar biopsy.^(49)^

### Vaginal cancer

In 2022, vaginal cancer accounted for 18,800 new cases and 8,238 deaths worldwide.^(5)^ The most important prognostic factor is the stage of the disease at the time of diagnosis, influenced by the size and depth of the lesion.^(50)^ The low survival rates compared to those of cervical cancer and vulvar cancer reflect the high proportion of diagnoses at advanced stages and potential complications of treatment in order to prevent more aggressive approaches.

For low-risk diseases (early stage, treated only with surgery and without adjuvant therapy), symptom assessment and physical examination are recommended every six months during the first two years and annually thereafter. For high-risk disease (advanced stage, treated with primary chemotherapy/radiotherapy or surgery plus adjuvant therapy), the frequency of surveillance may be every three months during the first two years, every six months between the third and fifth years, and then annually (Chart 7 and 8).^(5)^

**Chart 8:** Methods for following up patients after gynecologic cancer

	Endometrial cancer	High-grade and low-grade serous ovarian tumors and borderline tumors	Germ cell ovarian tumors	Stromal ovarian tumors	Neoplasms of the cervix, vulva, and vagina
Examination and review of symptoms					
Imaging tests			CT		
Tumor markers^a^	CA-125	CA-125	AFP Estrogen hCG LDH	AMH Estrogen Testosterone	
Patient cytology					

The color gradation from green to red corresponds to the indication of the follow-up methods for each neoplasm; green are the methods indicated, yellow are the methods indicated only if there is suspicion of recurrence and red are the methods not indicated. *Main tumor markers for germinal and stromal ovarian tumors. AMH: anti-Müllerian hormone; CT: computed tomography; AFP: alpha-fetoprotein; hCG: human chorionic gonadotropin; LDH: lactate dehydrogenase

Annual cervical or vaginal cytology tests, which may include HPV testing, can be indicated for the detection of lower genital tract dysplasia, although their value in detecting recurrent disease is limited and the likelihood of detecting asymptomatic recurrence is low. Furthermore, the accuracy of these tests may be affected in patients who have received radiotherapy, as it may induce changes in cell morphology.^(51)^

As in vulvar cancer, imaging and laboratory tests are recommended according to indications of suspicious findings on physical examination or symptoms of recurrence.^(52)^

In patients who have undergone radical surgery with neovagina, secondary cancer related to the technique used for reconstruction may occur. The neovagina should be examined by an experienced surgeon both during the surgical procedure and throughout subsequent follow-up. In patients undergoing chemoradiotherapy, the imaging method for assessing response to treatment should be the same as that used in the diagnosis and staging of the disease. The initial assessment of tumor response should not be performed before three months after completion of treatment. In case of uncertain response, reassessment should not be performed in less than eight to 12 weeks.^(51)^

## Final considerations

The follow-up of patients who survive gynecological tumors involves gynecologic oncologists and clinical oncologists. However, these professionals should not be the only ones involved. Dedicated general gynecologists, specialized nurses, general practitioners and a multidisciplinary team can also be involved in the follow-up of these patients. A comprehensive approach that includes education on signs and symptoms, side effects of treatment, the psychological consequences of the cancer diagnosis and functional losses, is extremely necessary. The assessment and support for the family and social needs should also be addressed. Common side effects secondary to previous treatments, such as sexual dysfunction and management of symptoms of premature ovarian insufficiency should also be assessed during follow-up. In this context, support through physiotherapy, sexual therapy and psychotherapy should be offered. In addition, the adoption of practices to improve the quality of life of these patients, such as the use of vaginal dilators and hormonal therapy should be evaluated when possible. It is important to provide counseling on genetic risk, guidance on fertility and contraception after treatment.

Psychological effects of cancer are common and can include depression, anxiety, fatigue, cognitive impairment, sleep problems, and opioid dependence. Assessing these symptoms is essential. Lifestyle changes, such as smoking cessation, healthy eating, regular exercise and moderate alcohol consumption are important and have shown to improve patients’ quality of life. In addition, the financial implications of treatment should be considered. Follow-up of patients treated for gynecological cancer may be a window of opportunity to promote screening for other primary neoplasms. Human papillomavirus infection is a risk factor for neoplasms of the cervix, vulva and vagina, anus, and oropharynx, which should be investigated during the evaluation, especially during the physical examination. Furthermore, with the increasing availability of genetic assessments before, during, or after cancer treatment, follow-up can be personalized based on information from the genetic profile. Patients treated for serous ovarian cancer with mutations in the BRCA1 or BRCA2 genes are at high risk of breast cancer and should be screened with mammography and breast MRI. Patients treated for endometrial cancer can be screened for Lynch syndrome, which would change the frequency of colorectal cancer screening.

Finally, it is important to consider that most of the evidence currently available is based on retrospective studies that cannot provide an accurate view of the benefits of recommendations. This points to the need for targeted research that can establish more cost-effective surveillance practices with proven effectiveness in detecting recurrences. Follow-up after treatment of gynecological tumors should focus on how care is provided to survivors. Regardless of the health resources available, it is crucial to ensure that patients receive the necessary support when they need it and that unnecessary follow-up is reassessed.
